# Mutually exclusive disorder-dependent hearing discomfort in first-episode psychosis and panic disorder: two experiments using the same auditory stimulus set and two similar musical sequences

**DOI:** 10.1186/s41155-022-00239-7

**Published:** 2022-12-08

**Authors:** Maria Lúcia de Bustamante Simas, Tamires Lima da Silva, Naianna Ribeiro Mocelin dos Santos, Aline Mendes Lacerda

**Affiliations:** Programa da Pós Graduação em Psicologia, Laboratório de Percepção Visual, Centro de Filosofia E Ciências Humanas, Universidade Federal de Pernambuco, Recife, 50670-901 Brazil

**Keywords:** Panic disorder, First-episode psychosis, Hearing discomfort, Hearing tolerance, Auditory sensitivity

## Abstract

We investigated the level of hearing tolerance in patients with first-episode psychosis (FEP) and panic disorder (PD) as compared to two different groups of healthy controls (HC, HC2), one for each experiment, because we used two distinct psychophysical paradigms. We evaluated auditory discomfort of 28 volunteers (14 with FEP and 14 HC) in the first study and of 42 volunteers (21 with PD and 21 HC2) in the second study. We presented 20 sounds: 16 pure-tone frequency sweeps (specially designed for use with FEP) and 11 s or 13 s musical sequences from the very beginning of the music “Play the Game” (PLAY) from Queen and its reverses. The first procedure used a Likert-like 0–10 scale ranging from “nothing bad” to “too bad” where volunteers made vertical marks along a horizontal line according to their discomfort. The second procedure involved subjective magnitude estimation online due to the SARS-COV-19 pandemic. Sounds were placed online and played by PD and HC2 volunteers themselves after having listened to the standard (the first 8 s from RADIO, “Radio Ga Ga” by Queen). Then, PD and HC2 volunteers were asked to assign values equal to, or multiples of 10 that *felt* like, or proportional to, their hearing “discomfort” in comparison with Sound 00 (RADIO). Our findings showed that FEP volunteers assign more discomfort to the 16 specially designed frequency sweep stimuli that appear not to affect HC, HC2, and PD. On the other hand, musical sequences from PLAY caused strong discomfort to PD in the reverse mode, but did not seem to affect HC, HC2, and FEP. Further experiments using the exact same paradigm with FEP and PD are needed to explore these findings.

## Introduction

Early detection of altered sensory and perceptual processes may be very helpful in preventing aggravation of psychiatric disorders. Most of these have the dangerous potential to impose severe handicap on people afflicted by them; thus, selective and accurate assessment to achieve precocious diagnosis are the main objectives to pursue.

Currently, the mainstream dealing with this sort of diagnosis focuses almost exclusively on testing cognitive executive functions and abilities. On the other hand, sensory measurements as potential biomarkers remain restricted to the helm of electrophysiological domain. Most of the methods that propose to assess sensory capabilities in first-episode psychosis (FEP), schizophrenia (SCHZ), panic disorder (PD), and the related illnesses do rely heavily on psychophysiological procedures like the mismatch negativity paradigm (MMN), or the auditory steady state response (ASSR), both requiring somewhat sophisticated equipment and data processing, items not so readily available in ambulatory/clinical general attendance.

Psychophysical methods can be very simple, direct, and easy to run in adequate ready-made experimental/clinical setups. Development and design of such resources could be highly advantageous in the initial assessment of people seeking help for potential neuropsychiatric disorders.

We have been working with the main assumption that sensory perception deteriorates faster and prior to the worsening of cognitive and executive functions in all psychiatric disorders (de Bustamante Simas et al., 2021, 2022). Hearing and vision are among the first to be affected, and, from our observations so far, hearing is the most precocious.

Searching the literature, we found no studies on hearing, or hearing sensitivity, using psychophysical methods and pure tone frequency 4–8 s sweep stimuli to assess people diagnosed, suspected or self-reported with FEP or PD. De Bustamante Simas et al. (2022) made an exhaustive review of the literature on hearing sensitivity in psychosis and introduced the idea of excessive, or enhanced, hearing sensitivity to specific pure tone frequency ranges in FEP and schizophrenia. In this study, we explore this idea further, suggesting the occurrence of increased hearing sensitivity to certain sound patterns in PD and possibly other neuropsychiatric disorders. So, in addition to pure tone frequency sweeps, we included sound patterns from an empirical observation of the complaint by a young 8-year-old child about how sinister the initial sequence of the music “Play the Game” (PLAY), from Queen, sounded like in his perception, and we decided to put it to test in our ongoing projects to run experiments with volunteers afflicted by FEP or PD.

In sum, we report here experiments with the use of two distinct psychophysical paradigms to evaluate auditory/hearing discomfort as an early sign of distress and therefore suitable to investigate the level of hearing tolerance in both patients with FEP and patients with PD. The first experiment was conducted with FEP volunteers and reported in part (for 16 of 20 sounds) by de Bustamante Simas et al. (2022). The second one, entirely reported here, was ran with PD and HC2 volunteers with the complete set of 20 sounds.

Both experiments used the 16 pure tone frequency 4–8 s sweep stimuli (specially designed for FEP and schizophrenia and reported by de Bustamante Simas et al., 2022), but used as well, two sequences from the very beginning of PLAY (lasting 11 s or 13 s), and their reverse (REVPlay) to estimate hearing tolerance. Thus, sound discomfort caused by this set of stimuli during individual presentation to volunteers diagnosed with FEP or PD, and matched healthy controls (HC and HC2, respectively) was measured with the use of two methods. For FEP, as reported by de Bustamante Simas et al. (2022), we used as psychophysical method the volunteers’ mark on a continuous Likert-like scale ranging from “nothing bad” to “too bad”, and for PD, because of the SARS-COV19 pandemic, we changed to the psychophysical method of subjective magnitude estimation from Stevens (1956) with online instructions and stimuli numbered from 00 to 20, where sound 00 was the standard, RADIO (a sequence from the initial 8 s of Radio Ga Ga, by Queen).

Since we used two distinct psychophysical procedures with FEP and HC, and PD and HC2, we knew that we could not directly compare the results between themselves, but we would be able to assess the differences between experimental groups and controls. In this case, our expectations were that FEP would report higher sound discomfort level (SDL), and PD higher subjective magnitude estimation of discomfort (SMD), for all stimuli, when compared to their respective controls.

## Method

### Participants

#### First-episode psychosis and healthy controls characterization

Twenty-eight volunteers participated in the study reported in part by de Bustamante Simas et al. (2022). Fourteen male volunteer (18–50 years old) patients from the ambulatory service PEP/HC/EBSERH/UFPE composed the first-episode psychosis group (FEP), and 14 mental health diagnostic-free participants tentatively matched to the experimental group for gender, age, and educational level composed the healthy controls group (HC). Table 1 reproduces FEP and HC characteristics (de Bustamante Simas et al., 2022).

**Table 1:** Group characteristics from FEP vs HC and PD vs HC2

Variables	HC *n* = 14	FEP *n* = 14	HC2 *n* = 21	PD *n* = 21	*p*^1^
Gender	0 female 14 males	0 female 14 males	15 females 6 males	17 females 4 males	
Age mean (SD)	27.86 (10.02)	25.57 (8.38)	23.81 (4.54)	30.29 (6.23)	0.491
Education					
Graduation incomplete	4	3	14	7	
Graduation completed	0	0	7	14	
High school incomplete	1	1	0	0	
High school completed	7	4	0	0	
Fundamental incomplete	1	4	0	0	
Fundamental completed	1	2	0	0	
Total	14	14	21	21	

Note: ^1^Mann-Whitney *U*-test

#### Panic disorder and healthy controls 2 characterization

Forty-two volunteers recruited online through social networks Instagram, Facebook, and WhatsApp, or through familiarity or friendship, were eventually contacted either by WhatsApp/phone, and/or email, for information and instructions on how to participate in the experiment (refer to Table 1 for sample characteristics). Twenty-one of them had panic disorder diagnose (19 by psychiatrists, two by psychologists and self-evaluation) and composed the PD group. The other twenty-one volunteers’ diagnostic-free of psychiatric illnesses composed the healthy control group (HC2). Table 1 shows PD and HC2 characteristics.

#### Assessment scales used for characterizing FEP and PD

Table 2 shows mean scores, standard errors (SE), and probability results from scales used for assessment of FEP vs HC and PD vs HC2.

**Table 2:** Group characteristics on assessment: mean scores, respective standard errors (SE), and probabilities for FEP vs HC and PD vs HC2

Variables	HC Mean (SE)	FEP Mean (SE)	HC2 Mean (SE)	PD Mean (SE)	*p*^1^
Addenbrooke’s cognitive examination, ACE-R (for FEP)	90.93 (1.84)	79.43 (3.55)	_	_	.009**
Beck Depression Inventory	_	_	36.05 (2.39)	49.62 (2.13)	.001***
Beck Anxiety Inventory	_	_	32.48 (1.88)	54.86 (3.16)	.001***
Stress Scale	_	_	45.14 (5.94)	76.52 (6.34)	.002**
Self-report of sensory-perceptual alterations — SRSPA^2^ (specially designed)	_	_	96.33 (17.91)	463.57 (51.79)	.001***

Note: ^1^Mann-Whitney *U*-test: *p > .05; **p > .001; ***p > .0001

As reported by de Bustamante Simas et al. (2022), both HC and patients diagnosed with FEP were tested only with the Addenbrooke cognitive examination, ACE-R (Amaral-Carvalho & Caramelli, 2007).

In the second experiment, patients diagnosed with PD and HC2 responded to the following scales:

(i) Online adapted and shortened version of Beck Anxiety Inventory, BAI (Beck et al., 1988; Cunha, 2001)
(ii) Online adapted and shortened version of Beck Depression Inventory, BDI-II (Beck et al., 1996; Cunha, 2001)
(iii) Stress scale (Benzoni, 2019)
(iv) Self-report of sensory-perceptual alterations inventory — SRSPA^1^ (a prototype developed by de Bustamante Simas & Lacerda for the study with PD patients).

### Instruments

#### Instruments for designing the auditory stimulus set and for selection and musical sequences from two songs by Queen

**Fig. 1:**
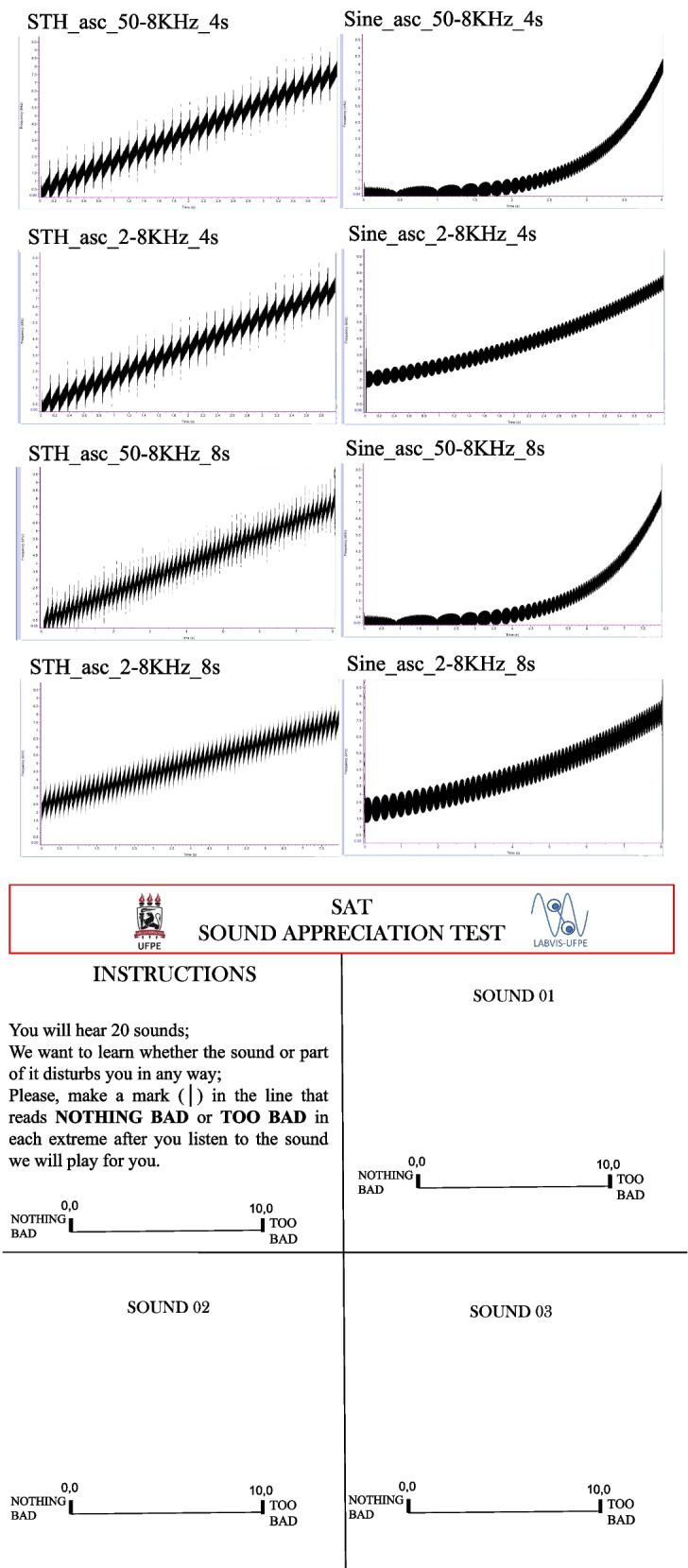
Spectra from the specially designed pure tone frequency sweep stimuli in the ascending mode **A** and sample from the response pad used by FEP and HC volunteers **B**. Note: From de Bustamante Simas et al. (2022)

(i) *Tone Generator/WavePad/MixPad NCH software* for sound creation, editing, and mixing (same as de Bustamante Simas et al., 2022).
(ii) Pure-tone frequency sweep stimuli for the sound appreciation test (SAT) produced with NCH software were as follows: 8 linear frequency sweeps with sawtooth (STH) envelopes (34 steps) and 8 logarithmic frequency sweeps with sine (SINE) envelopes (quasi-continuous, same as de Bustamante Simas et al., 2022).
(iii) The sixteen frequency sweeps were from 50 to 8000 Hz (*n* = 8), or from 2000 to 8000 Hz (*n* = 8), with durations of 4 s or 8 s each (*n* = 8, respectively), being 8 ascending (ASC) and the same 8 descending (DESC). Figure 1A shows spectra of the set of the eight ASC stimuli only (same as de Bustamante Simas et al., 2022).
(iv) Sequences from the very beginning of the song “Play the Game” (PLAY) from Queen with durations of either 11 s or 13 s and respective reverses (REVPlay).
(v) Sequence from the very beginning of the song “Radio Gaga” (RADIO) from Queen with duration of 8 s (assigned value 10 as standard in the subjective magnitude estimation method).

#### Instruments for experiments with FEP and HC (continuous Likert-like scale)

(i) Cell phone for sound stimuli presentation ~ 65 dB
(ii) Over-ear earphone JBL
(iii) Response pad with instructions, sounds numbered from 1 to 20, and lines sided by *nothing bad* on the left and *too bad* on the right side of a 10 cm horizontal line, according to Fig. 1B. Sixteen of these stimuli were reported by de Bustamante Simas et al. (2022).

#### Instruments for experiments with PD and HC2 (online subjective magnitude estimation method)

(i) Online stimulus set numbered 00 to 20 (on web page)
(ii) Online instructions to a magnitude subjective estimation procedure (on web page)
(iii) Stimulus 00 was a sequence lasting 8 s from the very beginning RADIO as the standard stimulus targeted as NOTHING BAD with the attributed arbitrary value of 10. This low attributed value is consistent with a one-sided (one tail) design.

## Procedures

### Procedure with FEP and HC [(this procedure has been reported for 16 of 20 stimuli in de Bustamante Simas et al. (2022)]

Upon consent from FEP ambulatory at Hospital das Clínicas/EBSERH/UFPE, Recife, PE, Brazil, submission to, and approval by the ethics committee (Plataforma Brasil-CAEE-n. 23,665,419.5.0000.8807), the research started.

After the experimenter assured full understanding of the procedure, the individual experimental session began always in the same sequence. Each participant signed the consent form and answered an interview about personal and medical background, followed by the ACE-R. Next, the sound appreciation test to evaluate hearing discomfort was also run individually and began by the instruction “You will hear 20 sounds. We want to learn whether the sound or part of it disturbs you in any way. Please, make a mark (│) in the line that reads NOTHING BAD or TOO BAD in each extreme after you listen to each sound, we will play to you.” After listening to each individual sound, volunteers made vertical marks along the horizontal line printed in a pad following a sequence of sheets numbered from 1 to 20 (refer to Fig. 1B), one sound number per sheet. The procedure lasted about 50 min. The last four sounds were sequences of PLAY and its reverse, not previously reported by de Bustamante Simas et al. (2022).

### Procedure using subjective magnitude estimation by PD and HC2

Research call for participants used the social media. Direct or spontaneous contacts through WhatsApp between volunteer and experimenter exchanging information about research objectives and requirements took place prior to any consent and approval granted by the ethics committee (Plataforma Brasil-CAEE N^∘^ 41,766,620.3.0000.5208).

Once agreed, the volunteer provided an email address to receive a link to Google Forms containing the consent form. If accepted, the experimenter sent editable documents in Word format with the BDI, BAI, and the stress questionnaire scales via WhatsApp/email.

The next step after answering the scales/questionnaires was to set a single appointment between participant and experimenter for a remote synchronous meeting (in the Google Meet platform) whose link was sent by email. In this meeting, the volunteer answered questions about background and familial history, answered the SRSPA scale, and, finally, was introduced to the experimental settings required for the SAT procedure.

For running SAT, volunteers wore earphones and set the volume at the level they normally listen to music while being directed to a web page with online instructions to listen to the sounds in the numbered sequence, starting from the standard, Sound 00, RADIO, assigned the value of 10. Volunteers played each sound themselves, in their own digital media. After listening to the standard, volunteers should assign values equal to, or multiples of 10 that *felt* to be like, or proportional, to their hearing “discomfort” in comparison with Sound 00 (RADIO). In the absence of discomfort, they should assign the value of the standard. Values attributed by the volunteers to each sound were spoken out loud following each presentation and immediately registered by the experimenter who was simultaneously online for the duration of the entire meeting. Upon completion of the procedure, the experimenter informed that participants would have access to results when available and showed appreciation for their participation. The complete meeting lasted about 40–60 min.

### Raw data handling with FEP and HC responses as reported previously

Real values within 0–10 cm attributed by the volunteers to estimate SDL were organized by stimulus modulation envelope (sawtooth, sine), order (ascending, descending), duration (4 s, 8 s), and range (0.050–8 kHz, 2–8 kHz), per group and per volunteer. We then run the *principal component analysis (PCA) followed by beta regressions* for each factor level to test the differences between groups (de Bustamante Simas et al., 2022).

### Raw data handling with PD and HC2 responses

**Fig. 2:**
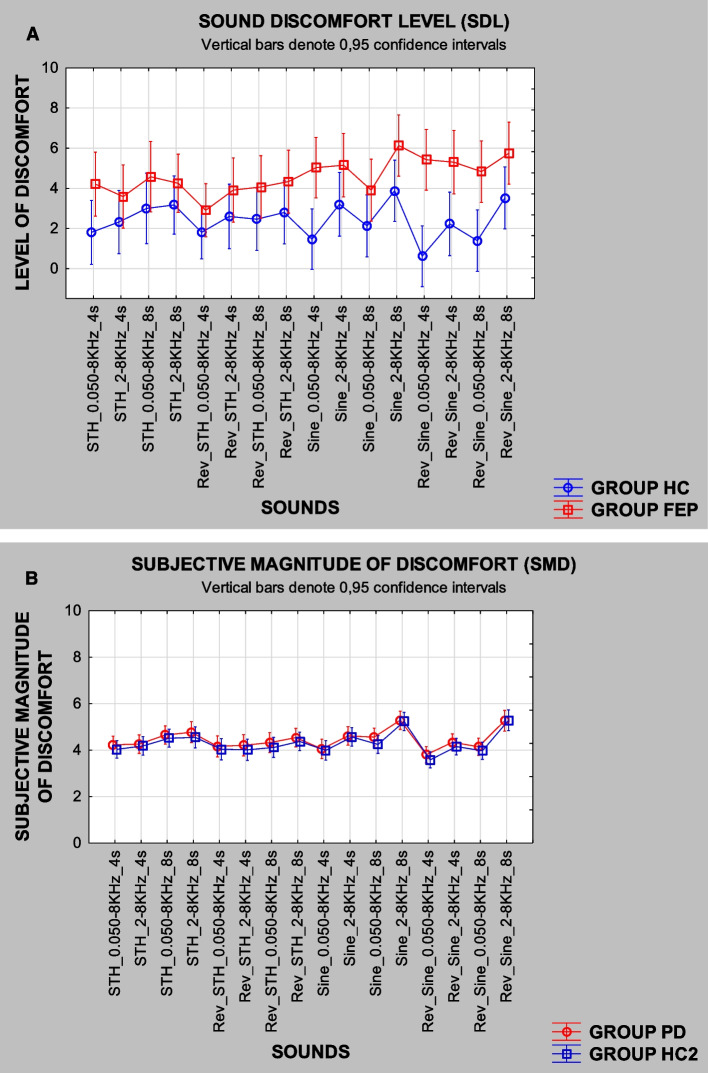
**A** Mean SDL observed for FEP and HC (after Fig. 4 from de Bustamante Simas et al., 2022) and **B** mean SMD observed for PD and HC2. Note: A variation of Fig. 4 from de Bustamante Simas et al. (2022)

After organizing the data in the same way as for FEP and HC, we transformed the data by calculating the logarithm of the volunteers’ responses following Fechner’s law (Fechner, 1860, 1966; Stevens, 1956, 1961) and normalized it by dividing the rounded maximum value of the whole sample so that values would be within (0, 1) interval for running beta regressions (due to small N size of the samples) to test for the differences between groups (PD vs HC2) for each the 20 individual sounds. We *chose not to do* linear fittings, nor to calculate the exponent from Steven’s power law (Stevens, 1956, 1961), because we were not estimating a sensory response that varied with the intensity of the stimuli. We were estimating the intensity of discomfort to stimuli that varied in frequency and not in amplitude as well as to sound streams of fixed amplitude interval, not varying systematically in amplitude from one stimulus to the next. Also, we did not do PCA analysis in the data for the 16 sounds from PD (as in de Bustamante Simas et al., 2022), because, as shown in Fig. 2B, these were very similar and barely differed from HC2.

## Results

### Beta regression analyses of FEP vs HC and PD vs HC2 data

Figure 2 reproduces the mean SDL attributed to the 16 pure tone frequency sweep stimuli by FEP vs HC (A) from de Bustamante Simas et al. (2022) and shows the subjective magnitude estimation of discomfort attributed to the same set of stimuli by PD vs HC2 (B). The logarithm of the raw values attributed to the sounds by PD and HC2 was divided by the maximum attributed magnitude of 3.4 as to yield values in the open interval between 0 and 1 to allow the use of the beta regression for group comparisons. Thus, in this Fig. 2, the resulting attributed values were multiplied by ten to set the same (proportional) arbitrary scaling values in both graphs: from FEP and PD. Despite the differences between procedures, the general profile of the attributed values by HC using Likert scales is very similar to those general profiles observed for PD and HC2 (that did not differ statistically) using subjective magnitude estimation.

As previously reported (de Bustamante Simas et al., 2022), six of the 16 sounds did differentiate FEP from HC as tested by the individual beta regressions run per stimulus (Table 3). Not only that, FEP consistently chose higher hearing discomfort values than HC in all cases (Fig. 2A). But, in the present study as we said, PD did not differ from HC2 in any of these very same 16 sounds (Fig. 2B); therefore, we did not run any beta regressions for PD and HC2.

**Table 3:** Beta regression coefficient estimates for FEP vs HC (*N* = 32), respective standard errors (SE), and *p*-values for all 8-factor levels used in designing the pure frequency sweep stimuli. Negative values merely reflect group order in spreadsheet (adapted from de Bustamante Simas et al., 2022)

Factor levels	*β* estimate	SE	*p*
STH SINE ASC DESC 0.05–8 kHz 2–8 kHz 4 s 8 s	− 0.277 − 0.325 − 0.313 − 0.301 − 0.214 − 0.278 − 0.266 − 0.338	0.135 0.138 0.133 0.142 0.152 0.136 0.156 0.144	0.040* 0.018* 0.019* 0.034* 0.160 0.041* 0.089 0.019*

Note: Based on beta distribution. *p < 0.05

On the other hand, both reverse sequences REVPlay that, by design, only differed in duration (11 s and 13 s) did differentiate (Table 4) between PD and HC2, *p* = 0.033 (11 s) and *p* = 0.015 (13 s), but did not differentiate FEP from HC (Table 4 shows results for FEP not published by de Bustamante Simas et al., 2022). Furthermore, the longer stream duration (13 s) yielded smaller *p*-value than the shorter stream duration in the case of PD vs HC2 (refer to Table 4).

**Table 4:** Beta regression coefficient estimates for FEP vs HC (*N* = 32) and PD vs HC2 (*N* = 42), respective standard errors (SE), and *p*-values

Musical sequences Play the Game	*FEP*			*PD*		
	*β estimate*	*SE*	*p*	*β estimate*	*SE*	*p*
PLAY 11 s PLAY 13 s REVPlay 11 s REVPlay 13 s	− 0.019 0.033 0.221 0.191	0.185 0.189 0.179 0.180	0.918 0.862 0.218 0.288	0.144 0.097 0.142 0.160	0.061 0.065 0.066 0.066	0.062 0.137 0.033* 0.015*

Note: Difference between groups *p < 0.05. Please note that these results for the musical sequences PLAY have not yet been published previously for FEP patients and are not reported in de Bustamante Simas et al. (2022)

### Alternative analysis using Kruskal–Wallis (ANOVA) with FEP vs HC and PD vs HC2 data

Another viable analysis involved the use of Kruskal–Wallis, nonparametric test with both FEP and PD. This analysis was less suitable for that data due to the small sample size. Nevertheless, SDL was significantly higher in the FEP for six sounds of frequency sweeps modulated by SINE envelopes: two ASC and four DESC. In other words, all DESC sound frequency sweep stimuli modulated by SINE envelopes provoked higher SDL (*p* < 0.05) in FEP than HC. So did two ASC sounds, namely, SINE, 0.050–8,000 Hz, 4 s, *p* < 0.05, and SINE, 2–8 kHz, 8 s, *p* < 0.05 (de Bustamante Simas et al., 2022). But, in the case of the sequences from PLAY and reverses, REVPlay, FEP did not differ from HC.

On the other hand, in the case PD vs HC2, SMD estimates were significantly higher for PD than HC2 only in the case of REVPlay 13 s, *p* < 0.029. Sequences PLAY 11 s and REVPlay 11 s, *p* = 0.0611 and *p* = 0.0651, respectively, did not reach significance levels. However, those probability results suggest the possibility of achieving significance level with an increased sample size. And, contrary to FEP and HC, no differences were found for the 16 pure frequency sweep stimuli between PD and HC2.

## Discussion

**Fig. 3:**
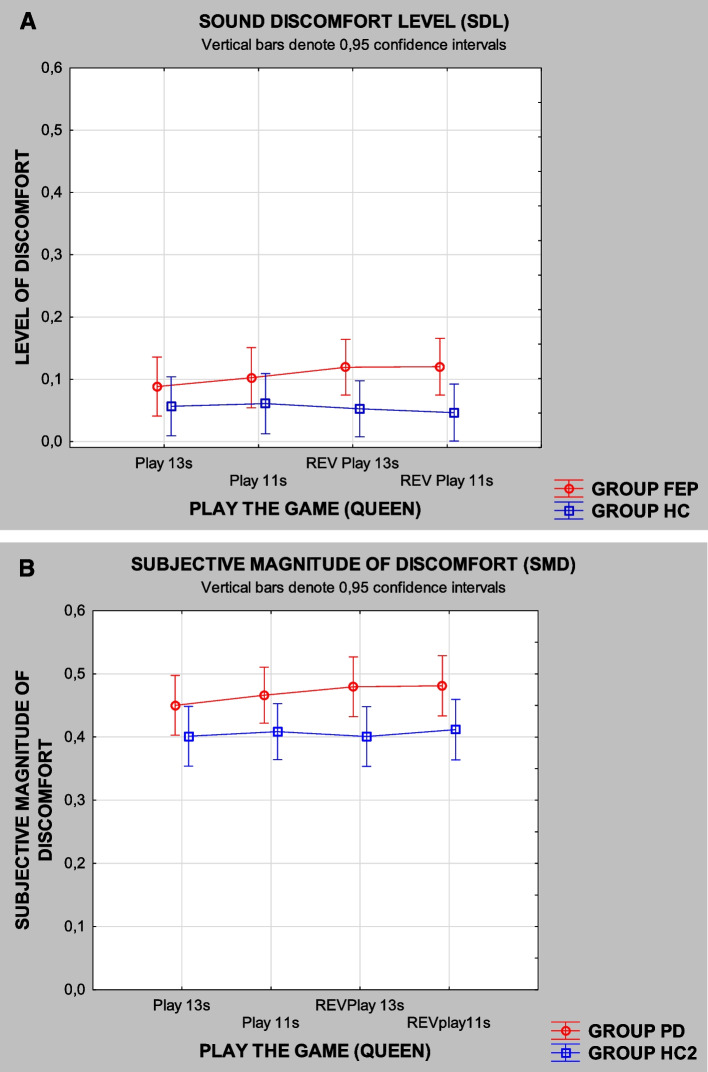
Mean SDL attributed to sequences from “Play the Game,” forward and reverse, by FEP vs HC **A** and mean SMD attributed by PD vs HC2 **B** to the same sequences

Our findings did not meet our earlier expectations; results from the analyses with beta regression to test differences between groups yielded quite unexpected outcome because hearing discomfort occurred in a selective and mutually exclusive fashion for FEP and PD. The 16 stimuli that seemingly caused discomfort to people with FEP in some way did not affect HC, HC2, and PD (Fig. 2). On the other hand, the sound streams from REVPlay strongly affected PD, but not HC, HC2, and FEP (Fig. 3).

Since we used two different paradigms, we cannot compare FEP and PD directly, but each procedure used controls that, as observed in Fig. 2 A and B, seem to produce similar and equivalent curve profiles. Unfortunately, we are also unable to compare values between the two procedures, but, in Fig. 3, both profiles, from controls and from experimental groups, are also similar: HC ~ HC2 and FEP ~ PD. Nevertheless, despite those similarities, beta regression analyses did not yield significant difference between FEP vs HC (*n* = 28) but did produce differences between PD vs HC2 (*n* = 42) for sequences from REVPlay lasting 11 s and 13 s (refer to Table 3). Using the alternative nonparametric Kruskal–Wallis ANOVA, only the sequence from REVPlay lasting 13 s differentiated PD from the other groups.

We assume these observed low hearing tolerance to some pure frequency sound sequences in the case of FEP, or some given musical sequence sequences in the case of PD, as early signs of distress disorder related. Indeed, we do find some support in the literature for this assumption. Findings of reduced sensitivity to changes in non-fear-related auditory stimuli as assessed by a MMN paradigm (Rentzsch et al., 2019), and of increased sensitivity to heartbeat sounds (considered as panic related by Zheng et al., 2019), as compared to neutral sounds in PD (also within a MMN paradigm) do support our findings. In our case, we found, with a psychophysical procedure, that the pure frequency sweeps would take the place of the neutral stimuli that do not disturb PD, while the reverse of the “sinister” beginning of PLAY would take the place of the fearful stimulus that causes discomfort to PD patients.

On the other hand, the enhanced auditory sensitivity of FEP finds support in the finding of Park et al. (2010), where they estimate loudness dependence of the auditory-evoked potential. They found schizophrenics to be less sensitive to increases in loudness (most likely due to excessive auditory sensitivity) than PD. In our design, loudness was not manipulated, pure frequency sweeps were, and these annoyed FEP but appear neutral to PD. But, in the “sinister” musical sequence PLAY and REVPlay, loudness oscillates and increases (or decreases), while frequencies disorderly vary intensities between 0 and 16 kHz, mostly around 0.5–8 kHz. Such sequences did affect PD, but our findings show that it did not differentiate FEP from HC.

In sum, we presented here two studies with two different psychophysical methods with two different populations, FEP and PD. We used different and specific psychological scales with the only intent of characterizing either FEP or PD samples. But we had to change our psychophysical procedure from one study to the next due to the SARS-COV-19 pandemic so that all experiments were done at a safe distance or online, remotely. Our findings did show difference between patients and controls in both cases, giving support to our selection and design of auditory stimuli and musical sequences that were the same in both experiments.

Thus, we strongly suggest the use of any of these two psychophysical procedures to assist in the assessment of individuals seeking neuropsychiatric help. Indeed, the series of screening tests we have been developing for use for that purpose include the present auditory experiments together with the visual experiments reported by de Bustamante Simas et al. (2021) and Lacerda et al. (2020). Not only that, but it also includes a test with dynamometer to assess handgrip force to estimate muscular strength that is weaken in acute psychotic states (Firth et al., 2018).

## Conclusion

This work presents two studies that compared auditory sound discomfort, or hearing tolerance, between FEP and HC and PD and HC2. It includes part of data and a variation of Fig. 4 from de Bustamante Simas et al. (2022) for the purpose of comparison. We made indirect comparison between FEP and PD and found inverse, and mutually exclusive, results for those neuropsychiatric disorders. Hearing pure frequency sweeps seem to be less tolerated by FEP than by PD and controls, while the musical sequences REVPlay appear to be less tolerated by PD than FEP and controls. Further experiments using the exact same paradigm with FEP and PD are needed to better explore the present findings.

## Data Availability

The datasets generated and/or analyzed during the current study are not publicly available due the rights of privacy regarding the volunteers for the experiments but are available from the corresponding author on reasonable request.

## Abbreviations

FEP: First-episode psychosis
PD: Panic disorder
HC: Healthy control group (matching FEP)
HC2: Healthy control group (matching PD)
PLAY: Musical segment from the song Play the Game by Queen
RADIO: Musical segment from the song Radio Ga Ga by Queen
ASC: Ascending order
*DESC: Descending order
*REV: Reverse order
STH_asc_0.050–8 KHz_4 s: Ascending sawtooth sweep envelop from 50 Hz to 8 kHz — 4-s duration
STH_asc_2–8 KHz_4 s: Ascending sawtooth sweep envelop from 2 to 8 kHz — 4-s duration
STH_asc_0.050–8 KHz_8 s: Ascending sawtooth sweep envelop from 50 Hz to 8 kHz — 8-s duration
STH_asc_2–8 KHz_8 s: Ascending sawtooth sweep envelop from 2 to 8 kHz — 8-s duration
Sine_asc_0.050–8 KHz_4 s: Ascending sine sweep envelop from 50 Hz to 8 kHz — 4-s duration
Sine_asc_2–8 KHz_4 s: Ascending sine sweep envelop from 2 to 8 kHz — 4-s duration
Sine_asc_0.050–8 KHz_8 s: Ascending sine sweep envelop from 50 to 8 kHz — 8-s duration
Sine_asc_2–8 KHz_8 s: Ascending sine sweep envelop from 2 to 8 kHz — 8-s duration
PLAY_11: Initial musical segment from Play the Game — 11-s duration
PLAY_13: Initial musical segment from Play the Game — 13-s duration
MMN: Mismatch negativity paradigm
ASSR: Auditory steady-state response
SDL: Sound discomfort level
SMD: Subjective magnitude estimation of discomfort level
PEP/HC/EBSERH/UFPE: FEP attending unit at the Hospital das Clínicas from Federal University from Pernambuco, Recife, PE, Brazil
BAI: Beck Anxiety Inventory
BDI-II: Beck Depression Inventory
SRSPA: Self-report of sensory-perceptual alterations inventory developed in our laboratory
SAT: Sound apperception test
OBS: DESC and REV refer to the same inverse sweep order. Whenever the order is reversed, REV is placed preceding the abbreviation

## Footnotes

1 This inventory was specifically designed to evaluate sensory and perceptual alterations addressing visual, auditory, olfactory, gustatory, and general bodily sensations as putative factors. The study with PD was the first to employ such scale that has successfully identified, and differentiated volunteers diagnosed with PD from healthy controls (HC2), but since it is not the main object of this study, its details will be reported elsewhere.
